# Treatment and outcomes in pheochromocytomas and paragangliomas: a study of 110 cases from a single center

**DOI:** 10.1007/s12020-018-1734-x

**Published:** 2018-09-15

**Authors:** Henrik Falhammar, Magnus Kjellman, Jan Calissendorff

**Affiliations:** 1 Department of Endocrinology, Metabolism and Diabetes, Karolinska University Hospital, Stockholm, Sweden; 2 Department of Molecular Medicine and Surgery, Karolinska Institutet, Stockholm, Sweden; 3 Department of Breast and Endocrine Surgery, Karolinska University Hospital, Stockholm, Sweden

**Keywords:** Incidentalom, Blood pressure, Diabetes, Mortality, Surgery, Metastasis

## Abstract

**Purpose:**

Many pheochromocytomas and paragangliomas (PPGLs) are nowadays diagnosed as incidentalomas or by screening. This may have changed outcomes.

**Methods:**

We reviewed 110 consecutive cases of PPGLs. Two cases with concurrent ectopic ACTH-syndrome were excluded.

**Results:**

Sixty-five percent had presented as incidentalomas, 30% as symptomatic PPGLs, and 5% had been screened (previously diagnosed MEN2A). Doxazosin was used in 79%, phenoxybenzamine in 18%, intravenous phentolamine in 1%, and no alpha-blockade in the rest. Laparoscopic surgery was performed in 70%, but 11% were converted to open surgery. Complications of surgery were seen in 20%, and length of stay after surgery was 4 days (2–8) with no correlation with alpha-blockade dose or time. In the whole cohort glycemic disturbances decreased by surgery (47% vs. 9%, *P* < 0.001). During 9.6 ± 7.2-year (median 8[4–13]) follow-up, 7% developed a new PPGL, 5% a PPGL-metastasis (KI67 > 2% *n* = 2; KI67 ≤ 1% *n* = 3; tumor size ≥ 95 mm *n* = 4), and 13% died (metastatic pheochromocytoma *n* = 2, hypertensive crisis *n* = 1, heart failure *n* = 2, other malignancies *n* = 5, and unclear *n* = 4). Surgery improved blood pressure and glycemic disturbances in the incidentaloma and the symptomatic PPGL. Recurrence was more common in the screening group. The symptomatic PPGL group was more likely to die of a PPGL-related cause. Surgery was more challenging in the paragangliomas, with less improvement in glycemic control than in the pheochromocytoma group. However, blood pressure and long-term outcomes were similar.

**Conclusion:**

The outcomes seemed slightly better than previous studies. Long-term prognosis was similar between pheochromocytomas and paragangliomas.

## Introduction

Pheochromocytomas and paragangliomas (PPGLs) are rare neuroendocrine tumors arising from the adrenal medulla or extra-adrenal paraganglia, respectively, which produce catecholamines [1]. PPGLs can present in many ways, and symptoms and signs can be difficult to interpret initially [2]. With the increasing use of imaging, more PPGLs are found as incidentalomas, i.e., masses found on imaging studies ordered for unrelated conditions [2]. In patients with adrenal incidentalomas, 0.6–4.2% are found to have a PPGL [3–5]. PPGLs are potentially fatal if not diagnosed and/or managed appropriately [6, 7].

Once diagnosed, the pre-operative treatment of choice is an alpha-blocker with progressive dose up-titration for at least 1–2 weeks before surgery to prevent peri-operative cardiovascular complications [6]. Laparoscopic surgery is usually recommended for smaller (<6 cm) pheochromocytomas and suitable paragangliomas, while open surgery is recommended for larger tumors, or if laparoscopic surgery fails [6]. Hypertension, paroxysmal or consistent, is very common and metabolic disturbances, such as diabetes, are fairly prevalent [1, 8]. In a large proportion of patients, hypertension has been reported to persist after surgery [9, 10], while metabolic derangements were more likely to improve [11]. Occasionally, both diabetes and hypertension resolve after surgery, although it is unclear how often. The histopathological diagnosis of PPGLs is typically straightforward, but discrimination between benign and malignant lesions is extremely difficult. The presence of metastasis is the only certain way to diagnose a malignant PPGL [12]. The prognosis of paragangliomas is considered worse compared to pheochromocytomas, and life-long follow-up has been suggested [1, 6].

The majority of PPGLs have previously been found due to symptoms of elevated catecholamines, but with increasing use of high-resolution imaging techniques, incidentalomas are becoming more common. We have recently reported that the majority of pheochromocytomas nowadays are found during the investigation of incidentalomas [2]. Moreover, family screening for genetic syndromes has increased, including those related to PPGLs (e.g., multiple endocrine neoplasia type 2 [MEN2A], Von Hippel Lindau syndrome [VHL], neurofibromatosis type 1 [NF1], and mutations in succinate dehydrogenase B, C, and D [SDHx]). Thus, PPGL treatment and outcomes could be expected to have changed over time and may not be as previous literature suggests.

The aims of the present study were to investigate in a large cohort of PPGLs the management, short-term (surgical complications, hypertension, and diabetes), and long-term outcomes (recurrence, metastasis, and mortality), and to determine if these are different depending on the mode of presentation, or tumor type.

## Materials and methods

Eligible for inclusion were all patients with an International Classification of Diseases version 10 (ICD-10) code of E27.5 (adrenomedullary hyperfunction) and/or C74.1 (malignant neoplasm of medulla of adrenal gland) being admitted and/or attending the outpatient clinic between June 2005 and January 2018 at the Department of Endocrinology, Metabolism and Diabetes, Karolinska University Hospital, Stockholm, Sweden. The patients’ electronic medical files were reviewed and if a PPGL could not be confirmed the case was excluded. The diagnosis was confirmed using both imaging and urine/plasma catecholamines, in addition to the histological result post surgery. At the time of the review, The National Population Register was also consulted to retrieve the date of death if applicable [13]. Attending physicians in Sweden code all hospital admissions and specialist outpatient visits with ICD-10 codes, and these are stored in both local and national databases [14]. The mode of presentation (incidentaloma, symptomatic PPGL [defined as a patient suspected to have PPGL due to symptoms and signs before biochemical confirmation and imaging], screening, mixed adrenaline and noradrenaline secretion, only noradrenaline secretion), final diagnosis (pheochromocytoma or paraganglioma), tumor size, peri-operative management, length of stay (from the day of surgery to discharge), histology (KI67 and suspicion of malignancy), blood pressure, glucose abnormalities (both at the time of PPGL diagnosis and at the first endocrine review 6–12 months after surgery), genetic results if found (many results were not automatically incorporated into the medical electronic files), follow-up time, mortality including cause of death, metastasis from the PPGL, and recurrence were noted. The screening group consisted of patients with a known familial disease that could cause PPGLs, and therefore underwent regular screening for PPGL. Catecholamines were measured using high-performance liquid chromatography (HPLC) for 24 h urinary adrenaline and noradrenaline (normal <80 and <400 nmol/24 h, respectively), and liquid chromatography–tandem mass spectrometry (LC/MS/MS) for plasma metanephrine and normetanephrine (normal <0.3 and <0.6 nmol/L, respectively). As not all individuals had had both tests performed, the highest urine or plasma level was divided with the upper level of normal and noted. Only noradrenaline secretion was defined as adrenaline and/or metanephrine levels being below the upper limit of normal. The definition suggested by Esienhofer et al. [15], i.e., noradrenergic tumors being defined by a tumor-derived increase in plasma normetanephrine with either a lack of increase in plasma metanephrine or an increase of metanephrine less than 5% that of both normetanephrine and metanephrine, could not be used since we did not have enough data on plasma metanephrines. The recorded blood pressures used in this study were measured with an appropriate-sized blood pressure cuff on two occasions at rest, mostly seated in an office or occasionally on the ward. KI67 was determined using standardized methodology in the clinically accredited pathology laboratory by staining with an anti-KI67 antibody and counting manually 2000 cells in hotspots. Evaluation of histology using the Pheochromocytoma of the Adrenal Gland Scaled Score (PASS) [12] or Grading System for Adrenal Pheochromocytoma and Paraganglioma (GAPP) was done in some cases, but not all. The definition of suspicion of malignancy was a PASS score ≥4, a GAPP score ≥3, or if none of these score systems had been used, if the pathologist wrote suspicion of malignancy and from the report it seemed likely that the score was ≥4 or ≥3, respectively. Improvement of blood pressure post surgery was defined as a reduction of systolic and diastolic blood pressure of at least 10 mmHg, together with and/or reduction in blood pressure medications. Prediabetes was defined as HbA1c 42–47 mmol/mol and/or fasting plasma glucose 6–6.9 mmol/L and/or random plasma glucose 7.8–11 mmol/L. Part of this cohort has been used in a previous study of the initial clinical presentation of pheochromocytomas [2].

The Regional Ethical Review Board in Stockholm, Sweden, approved the study and as this was a retrospective study, formal consent was not required.

### Statistical analysis

Mean ± SD (if normally distributed) or median and interquartile range (25–75%) were used. Two groups and continuous variables were compared with unpaired *t*-test (normally distributed) or Mann–Whitney rank-sum test, and three groups with one-way ANOVA (normal distributions) or with ANOVA on ranks test. In frequency table calculations, Chi-square or Fisher’s exact test were used, whichever was appropriate. Correlations were calculated using linear regression analysis. A *P* value <0.05 was considered significant. SigmaStat 3.0 for Windows (Systat Software Inc., San Jose, California) was used for all calculations.

## Results

In total, 110 cases of PPGLs were identified but two cases (one pheochromocytoma and one with adrenomedullary hyperplasia) had concurrent ACTH secretion and were therefore subsequently excluded from further analysis. These two cases have been described in detail previously [16]. Moreover, two patients with adrenocortical cancers and concomitant catecholamine excess but without an eligible ICD-code (E275 or C741) were known to us as well but were not included and have been presented elsewhere [17]. No head/neck paragangliomas were included. Thus, 108 cases were included in the analysis.

### All included patients with a PPGL

The mean age at diagnosis was 54.6 ± 17.3 years, 52% were females and tumor size was 45 (30–60) mm (Table 1). Median follow-up time was 8 (4-13) years and data were available on surgical complications (<30 days) on almost all patients (three had not had surgery, see below); blood pressure and glucose on all patients, if alive, at 6–12 months; and recurrence, metastasis, and mortality on all. A follow-up time of at least 5 years for determining recurrence, metastasis, and mortality was available on 78 patients (72%). At first presentation, none of the included patients had metastasis, and only one had multifocal disease. At diagnosis doxazosin was initiated and up-titrated in 85 patients (79%), phenoxybenzamine in 19 (18%), intravenous phentolamine in one (1%), and no alpha-blockade in three (3%; two patients had an unclear abdominal tumor which on histology turned out to be paragangliomas, of which one was converted to open surgery; one was “cured” by an acute necrosis of the pheochromocytoma and therefore had no alpha-blockade prior to surgery; none had any further surgical complications). All patients except three (3%, all pheochromocytomas) had surgery and the diagnosis were confirmed histologically. Of these three individuals, one refused surgery due to old age (77-year-old), one due to extensive ileal adenocarcinoma, and one deceased prior to surgery due to complications of multiple myeloma. However, both imaging and urine/plasma catecholamines clearly suggested a pheochromocytoma and they responded well to doxazosin. A clear majority had laparoscopic surgery (70%), of which 11% were converted to open surgery (Table 1). A fifth of the patients had complications of surgery (bleeding, infection, damage to other organs, and cardiovascular events [hypertensive crisis, heart failure including pulmonary edema, and stroke, all occurring during or within hours after surgery]). Length of stay after surgery was 4 days (2–8), with no correlation with alpha-blockade dose or time. After surgery, both systolic and diastolic blood pressure, as well as number of blood pressure medications decreased (*P* value all <0.001) (Table 2). Almost half had a glycemic disturbance at diagnosis (26% diabetes and 21% prediabetes, respectively), while less than a tenth had any disturbance after surgery (all diabetes, the majority on insulin, but all had improved glycemic control). In a fifth of all cases where the histology was available, there was some suspicion of malignancy, however, the KI67 (available in 97 cases) was low (Table 1). Genetic screening, analyzing a panel up to 15 different genes, was found in only a minority of patients (*n* = 32, 30%), of which 20 (59%) were negative and 12 (38%) positive (*NF1 n* = 3, *VHL n* = 3, *RET n* = 5, *SDHB n* = 1). However, in those with negative testing, variants of unknown significance were found in three individuals (*NF1*, *VHL*, and *MAX*). During the mean follow-up of almost a decade, 7% had recurrence of the PPGL, 5% a PPGL-related distant metastasis (all T2-T3, N0, M0 at surgery with four stage 2 and one stage 3 [18]), and 13% died. Of the five patients who developed PPGL-related metastasis (tumor size at adrenalectomy: 5.5, 9.5, 10.5, 10.8, and 12 cm, respectively), two (40%) had a KI67 >2% (3% and 12%, respectively) at surgery, while the other three patients (60%) had a KI67 ≤1%. Of the patients who did not develop PPGL-metastasis during follow-up, 18/92 (20%) had a KI67 of ≥2% (up to 10%), including two that were suspected malignant on histology. However, compared to patients without PPGL-metastasis with lower KI67, the patients with higher KI67 had shorter follow-up time (2.5[2–5] vs. 10[6–13] years, *P* < 0.001) and were younger (32[23–62] vs. 59[48–71] years, *P* = 0.002). They did not differ in terms of tumor size, new PPGLs, or number of deaths. Eight patients had a tumor size of 95 mm or more, of whom four (50%) developed PGGL-related metastasis, compared to one out of 99 (1%) with a tumor size less than 95 mm (*P* < 0.001). Of the 14 deaths in total, the cause of death was metastatic pheochromocytoma with multiple metastasis (*n* = 2, 14%), complications of hypertensive crisis (*n* = 1, 7%; died 20 days after adrenalectomy), heart failure (*n* = 2, 14%; one died 6 years after adrenalectomy; one died 30 days after the combined diagnosis of pheochromocytoma and amyloidosis secondary to myeloma, but due to severe heart failure could not have adrenalectomy), other malignancies (*n* = 5, 36%; breast *n* = 2, rectal *n* = 1, ileum *n* = 1, neck *n* = 1) and unclear (*n* = 4, 29%). However, none of the uncertain causes of death were likely to be PPGL-related and occurred at an age between 83 and 89 years. Thus, only two died within 30 days post surgery or diagnosis.

**Table 1:** Surgery and long-term outcomes in patients with pheochromocytoma or paraganglioma, also divided into mode of presentation

	All (*n* = 108)	Incidentaloma (*n* = 70)	Symptomatic PPGL (*n* = 33)	Screening (*n* = 5)	*P* value
Age at diagnosis (yrs)	54.6 ± 17.3	59.0 ± 15.6	48.8 ± 19.4	31.2 ± 8.8	**<0.001**
Females	56 (52%)	41 (59%)	11 (33%)	3 (60%)	*0.053*
Tumor size (mm)	45 (30–60)	46.5 (35–60)	41 (30–65.5)	20 (19–26.3)	**0.040**
U-A/P-MNE	2.9 (1.0–13.9)	3.0 (1.0–12.0)	2.7 (1.0–16.3)	2.7 (2.2–3.9)	0.994
U-NA/P-NMNE	6.5 (2.8–17.3)	7.3 (3.3–14.2)	8.7 (2.9–35.4)	1.5 (1.2–2.1)	**0.008**
Doxazosin dose preop (mg)	25.5 ± 13.5	24.8 ± 13.4	28.5 ± 14.4	20.0 ± 7.5	0.361
Phenoxybenzamine dose preop (mg)	55.0 ± 22.2	53.8 ± 24.5	56.5 ± 21.3		0.831
Days on alpha-blockage prior to sx	60 (33–91)	65 (38–93)	56 (31–86)	76 (40–90)	0.457
Surgery	105 (97%)^a^	67/70 (96%)^a^	33 (100%)	5 (100%)	0.433
Laparoscopic	73/105 (70%)	46/67 (69%)	22 (67%)	5 (100%)	0.310
Converted to open	8/73 (11%)	6/46 (13%)	2/22 (9%)	0 (0%)	0.638
Complications	21/105 (20%)	14/67 (21%)	7 (21%)	0 (0%)	0.518
LOS postop	4 (2–8)	4 (3–8)	5 (3.3–8)	2 (2–4)	*0.085*
Suspected malignant on histology	25/104 (24%)	13/67 (19%)	11/32 (34%)	1 (20%)	0.259
KI67 (%)	1.0 (1.0–1.4)	1.0 (1.0–1.0)	2.5 (1.0–4.3)	1.0 (1.0–2.4)	**0.019**
Follow-up (years)	9.4 ± 7.1	8.6 ± 6.1	11.5 ± 8.9	7.4 ± 4.7	0.129
New PPGL	8 (7%)	3 (4%)	2 (6%)	3 (60%)	**<0.001**
Metastasis	5 (5%)	2 (3%)	3 (9%)	0 (0%)	0.328
Dead	14 (13%)	8 (11%)	6 (18%)	0 (0%)	0.430

The patients screened due to familiar disease had a previously known *RET* mutation (MEN2A)

*PPGL* pheochromocytoma and paraganglioma, *yrs* years, *sx* surgery, *U* urinary, *P* plasma, *U-A/P-MNE* highest U-adrenaline or P-metanephrine level divided the upper level of normal, *U-NA/P-NMNE* highest U-noradrenaline or P-normetanephrine level divided the upper level of normal, *LOS postop* number of days admitted in hospital after surgery

Bold, *P* < 0.05. Italic, *P* = 0.05–0.09. *P* value evaluates the difference between the three last groups

^a^One declined due to old age, one due to separate extensive adenocarcinoma, and one deceased prior to surgery due to multiple myeloma

**Table 2:** Blood pressure and glycaemic abnormalities in patients with pheochromocytoma or paraganglioma, also divided into mode of presentation, at diagnosis and at the first endocrine follow-up outpatient visit after surgery

	All (*n* = 108)			Incidentaloma (*n* = 70)			Symptomatic PPGL (*n* = 33)			Screening (*n* = 5)			
	At diagnosis	Postop visit	*P*	At diagnosis	Postop visit	*P*	At diagnosis	Postop visit	*P*	At diagnosis	Postop visit	*P*	*P*(all)
Systolic BP (mmHg)	154 ± 29	124 ± 14	**<0.001**	152 ± 25	124 ± 12	**<0.001**	165 ± 35	126 ± 15	**<0.001**	129 ± 14	116 ± 9	0.123	**0.022/**0.318
Diastolic BP (mmHg)	89 ± 14	75 ± 9	**<0.001**	88 ± 12	75 ± 9	**<0.001**	93 ± 17	75 ± 11	**<0.001**	78 ± 9	80 ± 0	0.636	*0.084*/0.343
BP medication	1.0 (0–2.0)	0 (0–1.0)	**<0.001**	1.0 (0–2.0)	0 (0–1.0)	**0.017**	1.0 (0–2.0)	0 (0–1.0)	**<0.001**	0 (0–0)	0 (0–0)	1.000	**0.003**/0.263
Improvement BP		101 (94%)			65 (93%)			33 (100%)			3 (75%)		**0.003**
Diabetes	28 (26%)	10 (9%)	**0.020**	16 (23%)	6 (9%)	**0.037**	12 (36%)	4 (12%)	**0.044**	0 (0%)	0 (0%)	1.000	0.138/0.647
Diet only	11/28 (39%)	0 (0%)	**0.037**	7/16 (44%)	0 (0%)	0.121	4/12 (33%)	0 (0%)	0.516	0 (0%)	0 (0%)	1.000	0.194/1.000
OAD only	7/28 (25%)	3^a^/10 (30%)	1.000	3/16 (19%)	1/6 (17%)	1.000	4/12 (33%)	2^a^/4 (50%)	0.604	0 (0%)	0 (0%)	1.000	0.292/0.170
Insulin	9/28 (32%)	7^b^/10 (70%)	*0.062*	6/16 (32%)	5^d^/6 (83%)	0.149	3/12 (25%)	2^c^/4 (50%)	0.547	0 (0%)	0 (0%)	1.000	0.253/**0.022**
Prediabetes	23 (21%)	0 (0%)	**<0.001**	17 (24%)	0 (0%)	**<0.001**	4 (12%)	0 (0%)	0.114	2 (40%)	0 (0%)	1.000	0.321/1.000
Any glycaemic disturbance	51 (47%)	10 (9%)	**<0.001**	33 (47%)	6 (9%)	**<0.001**	16 (48%)	4 (12%)	**0.003**	2 (40%)	0 (0%)	1.000	0.616/0.647

The patients screened due to familiar disease had a previously known *RET* mutation (MEN2A)

Prediabetes was defined as HbA1c 42–47 mmol/mol and/or fasting plasma glucose 6–6.9 mmol/L and/or random plasma glucose 7.8–11 mmol/L

*PPGL* pheochromocytoma and paraganglioma, *BP* blood pressure, *Improvement BP* defined as reduction of systolic and diastolic BP at least 10 mmHg together and/or reduction in BP medications, *OAD* oral antidiabetic drugs

Bold, *P* < 0.05. Italic, *P* = 0.05–0.09. *P*(all) evaluates the difference between the three last groups

^a^One individual had reduced from 2 OAD to 1 OAD

^b^Three had reduced the daily requirement by >50%, and in all the glycaemic control was better

^c^One had reduced the daily requirement by >50%, and in both the glycaemic control was better

^d^Two had reduced the daily requirement by >50%, and in all the glycaemic control was better

### Comparisons between those presenting as an incidentaloma, symptomatic PPGL, or on screening

The majority (*n* = 70, 65%) of cases were found serendipitously, i.e., presented as an incidentaloma (all had a CT scan). In a third (*n* = 33, 30%), the PPGL was suspected before the biochemical confirmation and imaging. In a minor group (*n* = 5, 5%), regular biochemical screening for PPGLs had been implemented following diagnosis of a familial syndrome with an increased risk (all had a *RET* mutation, i.e., MEN2A). When comparing the three groups, some differences were evident. Those presenting as an incidentaloma were older, while those found on screening were younger (Table 1). There was a trend toward more men than women in the symptomatic PPGL group, while the opposite was seen in the other two groups. Tumor size was smallest in the screening group. No significant difference in the alpha-blockade dose was seen, though numerically the symptomatic PPGL group had the highest doses. In terms of surgery (collectively, laparoscopic, conversion to open surgery and complications), no differences were seen, although there was a tendency toward longer hospital stays post surgery in the symptomatic PPGL group, while the screening group had half or less number of days compared to the other groups (*P* = 0.085). Post surgery, both systolic and diastolic blood pressure, as well as number of blood pressure medications, decreased in the incidentaloma and symptomatic PPGL groups, but this was not evident in the screening group (Table 2). Similarly, improvements in diabetes and prediabetes were seen in the incidentaloma and symptomatic PPGL groups but not in the screening group. While the proportion of cases with histology suspicious of malignancy was not significantly higher in the symptomatic PPGL group, the group did have significantly increased KI67 compared to the other groups (Table 1). Mean follow-up time was similar between the three groups and the majority of the individuals in the screening group had recurrence of a pheochromocytoma in the other adrenal. In contrast, none of the individuals in the screening group were found to have a PPGL-related metastasis or died. The causes of death in the incidentaloma group were heart failure (*n* = 2, 25%), other malignancies (*n* = 4, 50%; breast *n* = 1, rectal *n* = 1, ileum *n* = 1, neck *n* = 1), and unclear (*n* = 2, 25%). In the symptomatic PPGL group, the causes of death were metastatic pheochromocytoma with multiple metastasis (*n* = 2, 33%), complications of hypertensive crisis (*n* = 1, 17%), other malignancies (breast *n* = 1, 17%), and unclear (*n* = 2, 33%). Patients with symptomatic PPGL were more likely to die of a clear PPGL-related cause than those who presented with an incidentaloma (3/4 vs. 0/6, *P* = 0.033).

### Comparisons between pheochromocytomas and paragangliomas

The age at diagnosis, gender distribution, presentation and alpha-blockade doses and time were similar between the two groups (Table 3). Tumor size was larger in the pheochromocytomas. Laparoscopic approach was less common in the paragangliomas compared to the pheochromocytomas, although this did not reach statistical significance (*P* = 0.101). The majority of patients with paragangliomas who had an initial laparoscopic procedure had to be converted to open surgery, and the length of stay after surgery was twice as long as for the pheochromocytomas. The proportion of cases with histology suspicious of malignancy and KI67 were comparable between the groups (Table 3). Post surgery, both systolic and diastolic blood pressure decreased similarly in the groups, but the number of blood pressure medications only decreased in the pheochromocytoma group (Table 4). However, the composite outcome improvement in blood pressure was similar. Improvements in diabetes and prediabetes prevalence were only seen in the pheochromocytoma group, but both groups demonstrated improved glycemic control in those with persistent diabetes post surgery. Mean follow-up time, recurrence of PPGL, and death were similar between the two groups, although no patients in the paraganglioma group were found to have a PPGL-related metastasis (Table 3). The cause of death in the pheochromocytoma group was metastatic pheochromocytoma with multiple metastasis (*n* = 2, 17%), complications of hypertensive crisis (*n* = 1, 8%), heart failure (*n* = 2, 17%), other malignancies (*n* = 5, 42%; breast *n* = 2, rectal *n* = 1, ileum *n* = 1, neck *n* = 1), and unclear (*n* = 2, 17%). In the paraganglioma group, the cause of death was unclear in both cases.

**Table 3:** Presentation, surgery, and long-term outcomes in patients with pheochromocytoma or paraganglioma

	Pheochromocytoma (*n* = 95)	Paraganglioma (*n* = 13)	*P* value
Age at diagnosis (yrs)	54.1 ± 17.9	58.6 ± 18.1	0.395
Females	47 (49%)	9 (69%)	0.298
Incidentaloma	60 (63%)	10 (77%)	0.506
Symptomatic PPGL	30 (32%)	3 (23%)	0.762
Screening	5 (5%)	0 (0%)	0.886
Tumor size (mm)	46.5 (30–63)	31 (22.5–46.3)	**0.030**
U-A/P-MNE	3.6 (1.3–14.8)	0.7 (0.4–0.9)	**<0.001**
U-NA/P-NMNE	6.0 (2.8–18.1)	7.5 (3.4–14.0)	0.961
Doxazosin dose preop (mg)	25.9 ± 13.3	22.5 ± 14.3	0.445
Phenoxybenzamine dose preop (mg)	52.9 ± 19.8	70.0 ± 42.4	0.324
Days on alpha-blockage prior to sx	60 (34–90)	75 (23–96)	0.892
Surgery	92 (97%)^a^	13 (100%)	0.803
Laparoscopic	67/92 (73%)	6 (46%)	0.102
Converted to open	4/67 (6%)	4/6 (67%)	**<0.001**
Complications	17/92 (18%)	4 (31%)	0.505
LOS postop	4.0 (2.0–7.3)	8.0 (6.0–18.3)	**<0.001**
Suspected malignant on histology	21/91 (23%)	4 (31%)	0.795
KI67 (%)	1.0 (1.0–1.5)	1.0 (1.0–1.2)	0.855
Follow-up (years)	9.6 ± 7.2	7.9 ± 6.8	0.435
New PPGL	7 (7%)	1 (8%)	0.601
Metastasis	5 (5%)	0 (0%)	0.886
Dead	12 (13%)	2 (15%)	0.870

The patients screened due to familiar disease had a previously known *RET* mutation (MEN2A)

*PPGL* pheochromocytoma and paraganglioma, *yrs* years, *sx* surgery, *U* urinary, *P* plasma, *U-A/P-MNE* highest U-adrenaline or P-metanephrine level divided the upper level of normal, *U-NA/P-NMNE* highest U-noradrenaline or P-normetanephrine level divided the upper level of normal, *LOS postop* number of days admitted in hospital after surgery

Bold, *P* < 0.05

^a^One declined due to old age, one due to separate extensive adenocarcinoma and one deceased prior to surgery due to multiple myeloma

**Table 4:** Blood pressure and glycaemic abnormalities in patients with pheochromocytoma or paraganglioma, at diagnosis and at the first endocrine follow-up outpatient visit after surgery

	Pheochromocytoma (*n* = 95)			Paraganglioma (*n* = 13)			
	At diagnosis	Postop visit	*P*	At diagnosis	Postop visit	*P*	*P*(all)
Systolic BP (mmHg)	155 ± 30	124 ± 30	**<0.001**	151 ± 19	123 ± 15	**<0.001**	0.669/0.780
Diastolic BP (mmHg)	89 ± 14	76 ± 9	**<0.001**	83 ± 9	72 ± 9	**0.004**	0.111/0.155
BP medication	1.0 (0.0–2.0)	0.0 (0.0–1.0)	**<0.001**	1.0 (0.0–2.0)	1.0 (0.0–1.3)	0.590	0.810/0.151
Improvement BP		88 (93%)			12 (92%)		0.601
Diabetes	27 (28%)	9 (9%)	**0.002**	1 (8%)	1 (8%)	1.000	0.207/0.762
Diet only	11/27 (41%)	0/9 (0%)	**0.034**	0 (0%)	0 (0%)	1.000	**0.007**/1.000
OAD only	7/27 (26%)	3^a^/9 (33%)	0.686	0 (0%)	0 (0%)	1.000	1.000/1.000
Insulin	8/27 (30%)	6^b^/9 (67%)	0.111	1/1 (100%)	1^c^/1 (100%)	1.000	0.532/1.000
Prediabetes	22 (23%)	0 (0%)	**<0.001**	1 (8%)	0 (0%)	1.000	0.360/1.000
Any glycaemic disturbance	49 (52%)	9 (9%)	**<0.001**	2 (15%)	1 (8%)	0.480	**0.031**/0.762

The patients screened due to familiar disease had a previously known *RET* mutation (MEN2A)

Prediabetes was defined as HbA1c 42–47 mmol/mol and/or fasting plasma glucose 6–6.9 mmol/L and/or random plasma glucose 7.8–11 mmol/L

*PPGL* pheochromocytoma and paraganglioma, *BP* blood pressure, *Improvement BP* defined as reduction of systolic and diastolic BP at least 10 mmHg together and/or reduction in BP medications, *OAD* oral antidiabetic drugs

Bold, *P* < 0.05. *P*(all) evaluates the difference between the two last groups

^a^One individual had reduced from 2 OAD to 1 OAD

^b^Three had reduced the daily requirement by >50%, and in all the glycaemic control was better

^c^HbA1c was halved by surgery (from 76 to 38 mmol/mol)

### Comparisons between mixed adrenaline and noradrenaline secretion vs. those with only noradrenaline secretion

Mixed adrenaline and noradrenaline secretion was present in 78 of 106 patients (74%), while the remaining PPGL only had noradrenaline secretion. Of those with only noradrenaline secretion, 18 (64%) were pheochromocytomas and the rest paragangliomas (*P* < 0.001). Only one paraganglioma had mixed secretion (*P* < 0.001 compared to pheochromocytomas). Comparing those with mixed secretion vs only noradrenaline secretion, the former were older at diagnosis (57.1 ± 16.1 vs. 46.5 ± 20.7 years, *P* = 0.007), had higher adrenaline secretion (5.6[2.4–18.3] vs. 0.7[0.7–0.8] times the upper reference limit, *P* < 0.001), and had more diabetes at diagnosis (35% [*n* = 27] vs. 4% [*n* = 1], *P* = 0.003), with more oral diabetes medicine treatment (30% [*n* = 8] vs. 0% [*n* = 0], *P* = 0.002). Of those with metastasis, four had mixed secretion and one had only noradrenaline secretion (*P* = 0.852). All the other comparisons were not statistically different (data not shown).

## Discussion

This large study reinforces some results of previous studies but also demonstrates novel findings, including that mode of presentation may predict outcomes. Blood pressure and glycemic abnormalities improved in most patients, but recurrence, PPGL-related metastasis, and PPGL-related deaths still occurred, confirming the necessity for long-term follow-up.

Retrospective studies have shown that alpha-blockers are the preferred choice to reduce peri-operative complications [6]. Phenoxybenzamine is a long-acting, non-selective alpha-blocker which has traditionally been used, but short-acting selective alpha-blockers, such as prazosin, terazosin, and doxazosin, are becoming more popular due to fewer side-effects [6]. The vast majority of our patients were on doxazosin which was up-titrated to doses sometimes higher than the recommended final dose of 32 mg/day [6]. The dose or time on alpha-blockers did not differ between how the patient presented or between the pheochromocytomas and paragangliomas. Laparoscopic approach was the standard approach in all groups except in the paraganglioma group. However, while almost half of the paraganglioma group had an initial laparoscopic approach, two-thirds of them required conversion to an open procedure and the length of hospital stay post surgery was twice as long. Laparoscopic surgery for paragangliomas has been claimed as safe with outcomes similar to laparoscopic surgery for pheochromocytomas [19]. In contrast, our results indicate that a laparoscopic approach should be used only in select patients, which is in accordance with international guidelines [6]. Otherwise, aside from a tendency to longer post-operative stays in the symptomatic PPGL group, no differences were found between the different groups concerning peri-operative outcomes.

Hypertension, both sustained and paroxysmal, is common in PPGLs, with up to 90% affected at diagnosis [1, 2, 20]. Hypertension in PPGL has been considered potentially curable with surgery. We found a dramatic drop in blood pressure with surgery for all PPGLs, with the majority being able to cease all blood pressure medications and having normalized blood pressure postoperatively. The drop was similar in all subgroups, except for those found by screening, probably due to normal blood pressure at diagnosis. However, the paraganglioma group received the same amount of antihypertensive medication post surgery as at diagnosis, despite normalization of blood pressure. Our improvement in blood pressure seems much better than others who have shown persistent hypertension in 51–79% at last follow-up [9, 10]. Weismann et al. [10] reported that only 38% had improved blood pressure using similar criteria as us, compared to 94% of our patients. On the other hand, our blood pressure follow-up was done 6–12 months post surgery, and we did not have long-term follow-up data of blood pressure. Our patients were probably not as symptomatic, since less than a third presented with suspicion of PPGL, compared to 57 to >85% reported by the other research groups [9, 10, 20]. Nevertheless, all our patients in the symptomatic PPGL group also had blood pressure improvement by surgery. It has been reported that of 46 patients with hypertension after PPGL surgery at long-term follow-up, 30 (65%) were already hypertensive at 1 year post surgery. In our 6–12 months follow-up data, the majority were normotensive, suggesting that PPGL surgery may cure hypertension in most patients nowadays.

Glycemic disturbances are common at PPGL diagnosis [1, 11, 20, 21]. We found 26% of our patients with PPGL were affected by diabetes, which is slightly less than the 40% given in reviews [1], but similar to the 31% found in the most recent included patients by Amar et al. [20], and in a recent Japanese study [21]. Interestingly, in the last study, all patients underwent an oral glucose tolerance test (OGTT) and 4 of 13 (31%) included patients had impaired glucose tolerance, compared to our 21%. In contrast, we did not perform regular OGTTs but diagnosed prediabetes on fasting, random glucose levels, and/or HbA1c, which likely explains this difference. Of note, our symptomatic PPGL group had a higher frequency of diabetes compared to the incidentaloma or screening groups, but the latter two groups had more prediabetes, such that overall glycemic disturbances were similar. It could be speculated that the shift in presentation of PPGL will ameliorate the glycemic disturbances at presentation even more in the future. Like other studies, we found a dramatic improvement in insulin sensitivity by surgical treatment [11, 21], and most of our patients had no signs of diabetes post surgery, especially those on diet control preoperatively. Those with persistent diabetes could mostly reduce their insulin doses by >50%, or decrease the number of oral antidiabetic drugs, while still maintaining improved glycemic control. Thus, surgery in PPGL can lead to resolution of diabetes, or at least dramatically improve glycemic control. Interestingly, in our study the prevalence of glycemic disturbance was almost four times more common in the pheochromocytoma group compared to the paraganglioma group, and the latter group did not improve much by surgical treatment. Such a comparison has never been made previously [8]. Adrenaline has been reported to impair glucose metabolism more than noradrenaline [8]. Since functional paragangliomas almost exclusively produce noradrenaline, this could explain the difference.

The histological discrimination between benign and malignant tumors is challenging, with metastasis being the definition of a malignant PPGL according to WHO [12]. By this definition, 5% of our cases were malignant, with almost twice that number in the PPGL suspicion group. Around a forth of our cases were suspected malignant. The fact that not all cases were assessed formally using the PASS or GAPP scores could be viewed as a limitation, however, scoring systems have not been found to be reproducible and reliable, and their utility in predicting future metastasis has not been proven [12]. KI67 is a marker being used in many tumors for predicting malignancies. Most of our patients had very low KI67, but the symptomatic PPGL group had a median value of 2.5%. One study reported that two out of four cases with a KI67 >2% had future metastasis, compared to three out of 41 with lower KI67 [22], which is similar to our findings. Thus, although KI67 index may be useful as a risk estimator at group level, it is not valuable to predict metastatic development in individual patients given the large number of false-positive and false-negative results. In contrast, tumor size seemed more valuable in predicting future metastasis with a cutoff value of 9.5 cm in our study. In guidelines, the importance of size for the prediction of metastasis have been emphasized [6, 23].

The long-term outcomes appear reasonable in our study, with 13% having either recurrence or metastasis. This is slightly lower than others have reported [20], although when they divided their PPGL cohort into quartiles based on date of operation, patients in the most recent quartile (surgery around 2000) had similar figures to ours. In a previous similar study, metastasis, but not cardiovascular disease, was reported to reduce life expectancy [24]. Of the 64 patients with presumed benign PPGL, seven (11%) developed PPGL-related metastasis during follow-up, and all died. In our cohort, only two of the five developing PPGL-related metastasis died, five of the total cohort died of other malignancies, and three cardiovascular deaths occurred, of which two probably were at least partly PPGL-related. However, in almost a third of the deaths in our study, we did not identify a cause of death, which is similar to the previously mentioned study [24]. Thus, the long-term mortality in PPGL seems to predominately be due to PPGL-related metastasis and other malignancies, but longer follow-up with better documentation of the cause of death is needed to evaluate this further.

The only significant difference in long-term outcomes between the different subgroups in our study was an increased recurrence rate in the screening group, which is in accordance with others [20]. However, looking at the absolute numbers, the symptomatic PPGL group seemed to have a slightly worse prognosis, but the numbers were small. Moreover, the cause of death in the symptomatic PPGL group was 50% PPGL-related, while none in the incidentaloma or screening group had a PPGL-related death. This pattern has not previously been investigated. In contrast to what is normally stated in review articles and guidelines [1, 6], we could not demonstrate any worse prognosis in paragangliomas compared to pheochromocytomas. This was encouraging, but it should be noted that we only included paragangliomas with catecholamine excess due to the ICD-10 codes used, i.e., nonfunctional paragangliomas were excluded since they usually receive other ICD-10 codes. Moreover, the paraganglioma group was quite small so the study may have been underpowered to show a difference.

Almost all our paragangliomas secreted only noradrenaline but most pheochromocytomas had mixed adrenaline and noradrenaline secretion, which is similar to others [24, 25]. The outcomes between mixed and only noradrenaline secreting tumors were not different, however, those with mixed secretion were older and had higher rates of diabetes at presentation.

Like all retrospective studies, there are several limitations, in particular that of ascertainment bias. Not all cases had had a genetic evaluation. While this is a large study compared with similar single center studies, some subgroups were quite small, thus results from these subgroups must be interpreted with caution. Furthermore, we were not able to standardize the measurements and follow-up due to the retrospective nature of the study, however, the follow-up of mortality was complete thanks to the comprehensive coverage of The National Population Register.

In conclusion, the mode of presentation may be associated with short- and long-term outcomes. Our outcomes overall seem slightly better than previous studies. Short-time outcomes were slightly better for pheochromocytomas compared to paragangliomas, but long-term outcomes were similar. All PPGLs benefit from treatment, but earlier diagnosis may be better. Long-term follow-up is necessary [23].
